# Geospatial dataset on deforestation and urban sprawl in Dhaka, Bangladesh: A resource for environmental analysis

**DOI:** 10.1016/j.dib.2025.111786

**Published:** 2025-06-12

**Authors:** Md. Fahad Khan, Md. Rakibul Islam, Shanto Kumar Basak, Ahmed Imtiaz, Abhijit Bhowmik, Dip Nandi, Mashiour Rahman, Debajyoti Karmaker

**Affiliations:** Department of Computer Science*,* American International University-Bangladesh*,* Dhaka 1229*,* Bangladesh

**Keywords:** Computer vision, Deep learning, Image classification, Semantic segmentation

## Abstract

This dataset comprises high-resolution satellite images for monitoring deforestation in Dhaka, Bangladesh. Data were acquired via Google Earth Pro from fixed locations to maintain consistency in observing tree cover alterations. Each image includes annotations and JSON mask files that delineate tree cover and deforested areas. The dataset facilitates machine learning applications, including object detection, semantic segmentation, and change detection. Image resolution and aspect ratio vary, with 5-35 images recorded per location annually over a decade. This data serves researchers investigating urbanization's environmental impact and the gradual reduction of tree cover in a rapidly evolving urban environment. Utilizing the annotations and masks enables the training of machine learning models to identify and forecast vegetation changes, aiding environmental monitoring and conservation initiatives. Furthermore, the dataset is readily applicable for educational purposes in disciplines such as geography, environmental science, and machine learning. It provides critical insights into the application of machine learning and image processing in addressing real-world environmental issues.

Specifications Table

**Table:** 

Subject	Remote sensing, deforestation analysis, and environmental science.
Specific subject area	Computer Vision, Image processing, Deep learning
Type of data	Images, JPG format; JSON files with annotations and masks.
Data collection	Google Earth Pro facilitated the acquisition of satellite imagery to monitor deforestation in Dhaka, Bangladesh. Multiple years of images were systematically captured from specific locations, allowing comprehensive analysis of tree cover reduction. The imagery displays diverse aspect ratios based on satellite perspectives and possesses high resolution, suitable for remote sensing. Each site provided 5 to 35 images annually, accumulating data over a ten-year period. The dataset classifies images into three primary categories: tree cover, deforested regions, and masked images. Organized by year, it comprises both raw and annotated images, each paired with a JSON file containing annotations and segmentation masks. This organization enhances accessibility and temporal analysis. Furthermore, the dataset is conducive to machine learning initiatives, particularly in training models for object detection and segmentation to evaluate environmental alterations.
Data source location	Google Earth Pro Dhaka, Bangladesh
Data accessibility	The dataset is published in Mendeley Data. • Data identification number(doi): 10.17632/hst78yczmy.5 • Direct URL to data: https://data.mendeley.com/datasets/hst78yczmy/5

## 1 Value of the Data

The dataset provides insights into deforestation trends in Dhaka, Bangladesh, and invaluable asset for advancing research and education at the intersection of technology, environmental science, and sustainable development.

• Provides detailed insights into deforestation trends in Dhaka, Bangladesh, enabling analysis of urbanization's impact on tree cover.
• Serves as a valuable resource for researchers studying environmental and urban changes over time.
• Includes annotated images with JSON masks, ideal for training machine learning models in object recognition, classification, and segmentation in environmental and geospatial contexts.
• Bridges environmental monitoring with AI-driven solutions, supporting machine learning applications in ecological conservation and sustainability studies.
• Offers an adaptable structure for educational purposes, supporting coursework in geography, remote sensing, and machine learning.
• Allows students to explore the intersection of technology and environmental science, fostering practical skills in remote sensing and ecological impact analysis.

## 2 Background

Deforestation occurring within the geographical boundaries that delineate Dhaka, Bangladesh, is primarily driven by a complex interplay of various factors. The phenomenon characterized by the incessant and unyielding proliferation of urban territories, the persistent and continuous increase in population density, along with the swiftly advancing tempo of industrialization, collectively engenders considerable and profound alterations in land use and land cover (LULC) patterns that are evident throughout the entire region. During the temporal span from 1990 to 2020, there has been a remarkable increase of 188.35% in the extent of built-up areas, concurrently with the transformation of agricultural lands and aquatic ecosystems into developed spaces, which has precipitated a dramatic reduction of 59.55% in the value of urban ecosystem services, thereby translating into an economic detriment estimated at approximately 85 million USD. [2]. Additionally, illegal logging and land conversion for commercial activities exacerbate the situation, particularly affecting the Sal forests [3]. The lack of effective land management and enforcement of environmental protection laws further complicates these issues [1,4]. Consequently, these factors not only threaten biodiversity but also disrupt local ecosystems, highlighting the urgent need for integrated policy measures to mitigate deforestation impacts [4]. While the immediate focus is often on urban development, it is crucial to recognize that sustainable practices and effective policy implementation are necessary to mitigate the adverse effects of deforestation on Dhaka's ecosystem. Semantic segmentation in satellite images significantly enhances the accuracy of deforestation mapping in tropical regions by leveraging advanced deep learning techniques. The integration of models such as U-Net and its variants has demonstrated remarkable performance in delineating forest cover, achieving accuracies exceeding 95% in various studies [5,7]. These models effectively process complex spatial data, allowing for the identification of deforestation patterns amidst challenges like cloud cover, which is prevalent in tropical climates [6]. Furthermore, the employment of time series satellite imagery serves as a crucial tool that allows for the meticulous identification and analysis of dynamic and often complex alterations in forest cover, thereby significantly enhancing our comprehension of the fundamental factors and underlying mechanisms that contribute to the phenomenon of deforestation. [8]. Recent innovations, such as the Sharpened Cosine Similarity U-Net, further optimize segmentation processes, reducing training time while maintaining high accuracy [9]. Collectively, these advancements underscore the critical role of semantic segmentation in fostering sustainable forest management and conservation efforts.

## 3 Data Description

**Fig. 1:**
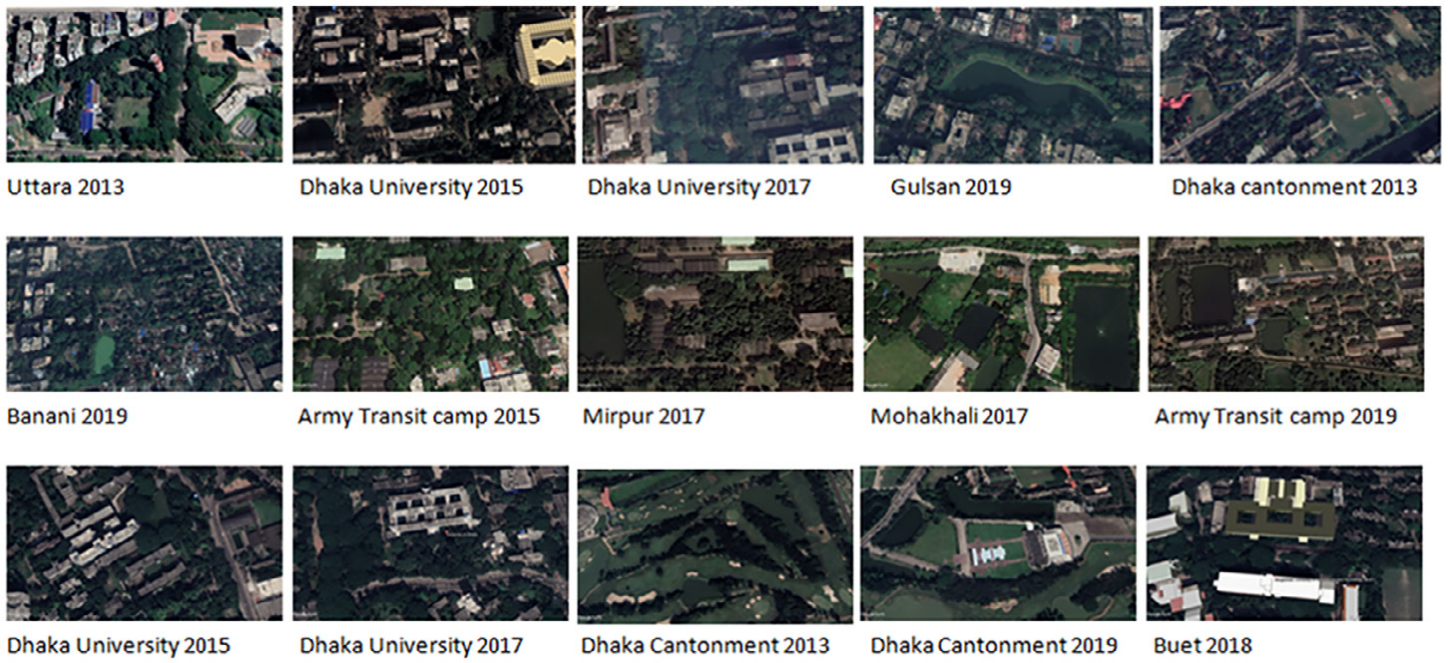
Sample of original image.

This dataset consists of satellite images categorized into two main types of regions in Dhaka with high tree cover and areas that have experienced significant deforestation. The images showcasing high tree cover represent areas that have largely been spared from deforestation, serving as reference points for understanding baseline environmental conditions and facilitating comparisons for tracking vegetation changes over time. These images are essential for modeling healthy ecosystems and for distinguishing between forested and deforested regions in machine learning tasks (see Fig. 1 for a sample of the original picture).

**Fig. 2:**
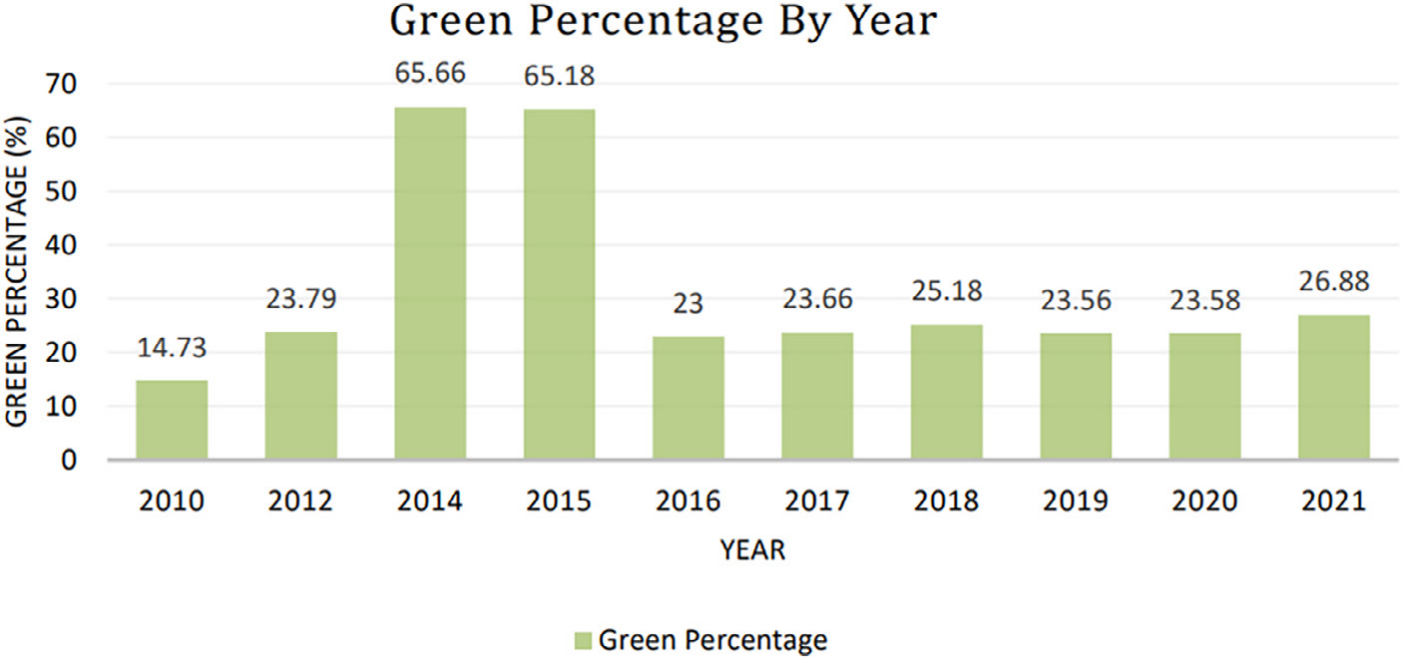
Green percentage from 2010 to 2021.

The dataset also includes a detailed analysis of the green percentage over the years, highlighting the changes in tree cover and deforestation trends. The following Fig. 2 illustrates the year-wise green percentage from 2010 to 2021, providing a clear visual representation of the decline in green cover over time.

This visualization underscores the significant reduction in green cover, particularly between 2010 and 2014, and the subsequent stabilization at a lower level. The data is crucial for understanding the long-term environmental impact of urbanization and deforestation in Dhaka.

In contrast, the images highlighting deforested areas clearly mark the regions where a large portion of tree cover has been lost, with corresponding masks delineating the extent of tree loss. These masks serve as baseline data for segmentation and detection tasks, enabling accurate analysis of deforestation rates and trends over time. The annotated regions help identify patterns of environmental degradation and contribute to conservation efforts.

**Fig. 3:**
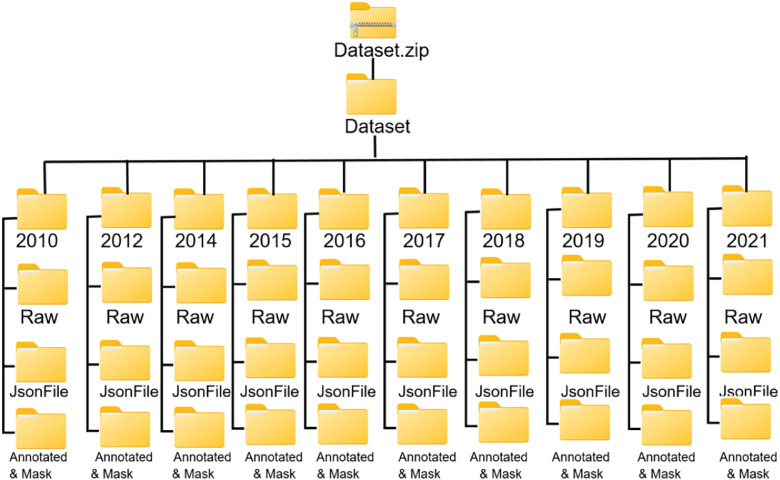
Data structure of the dataset.

Each visual representation within the dataset is supplemented by a mask file formatted in JSON, which encompasses pixel-level delineations of particular regions of significance. These masks identify various features, such as tree cover and deforested regions, making the dataset suitable for tasks like semantic segmentation, object detection, and change detection. The configuration of the dataset's data structure is meticulously crafted to facilitate seamless navigation and utilization. Fig. 3 for an illustration of the data structure. The masks can be directly utilized for training AI models, enabling automated analysis of landscape changes and aiding in the development of deforestation prediction algorithms. This structured approach not only enhances the understanding of environmental dynamics but also supports proactive conservation strategies.

## 4 Experimental Design, Materials and Methods

**Fig. 4:**
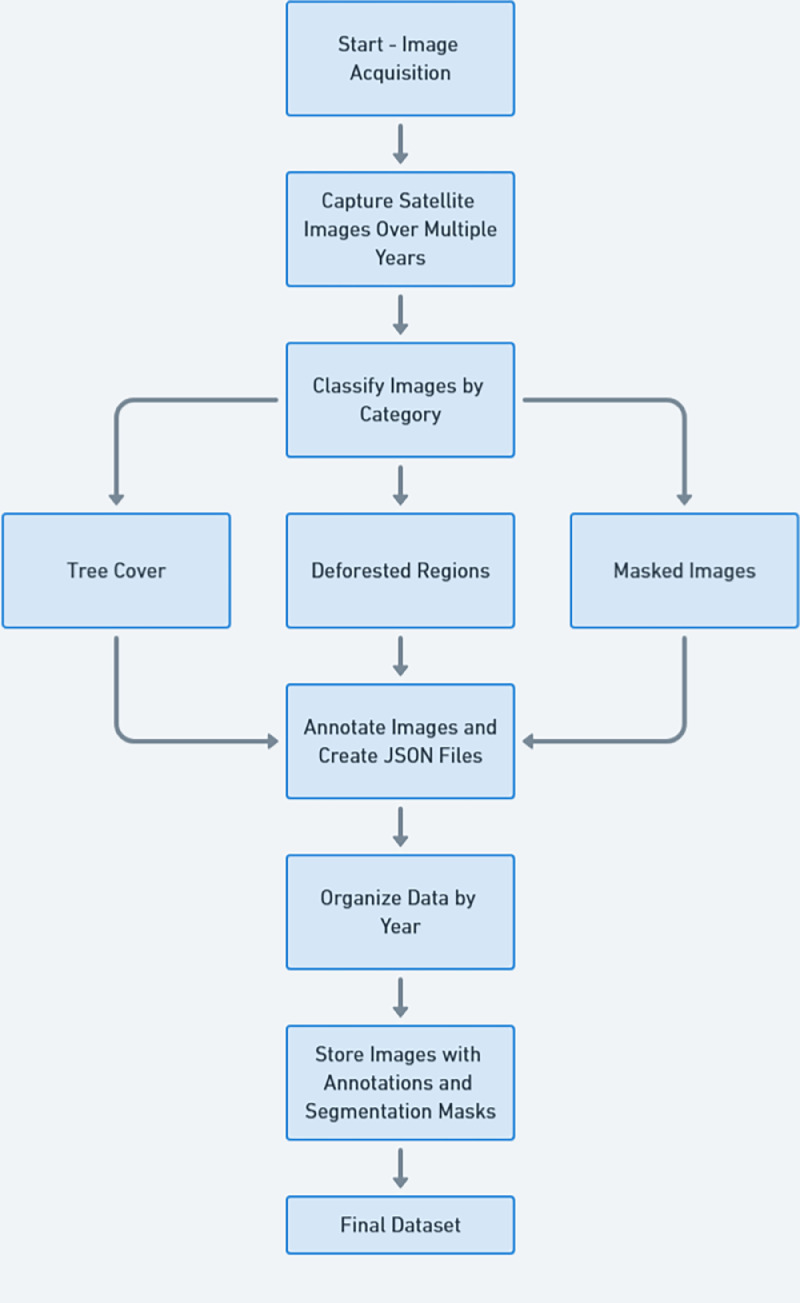
Workflow of the dataset creation.

The general experimental design and dataset creation methodology are covered in this section. Fig. 4 illustrates this process.

### 4.1 Data collection

Google Earth Pro was utilized to capture satellite imagery for the purpose of monitoring deforestation patterns in Dhaka, Bangladesh. Over an extended period, these images were acquired from strategically selected positions throughout the capital, facilitating a comprehensive analysis of the progressive degradation of arboreal coverage in the area. The acquired images exhibit diverse aspect ratios corresponding to the satellite perspective and uphold a high resolution conducive to remote sensing evaluation. On an annual basis, each site produced between 5 to 35 images, with data amassed over a span of ten years. The dataset categorizes the images into three main types such as tree cover, deforested areas, and masked images. Systematically arranged into annual folders, the dataset comprises both unprocessed and labelled images, with each image accompanied by a JSON file that contains annotations and segmentation masks. This organizational framework permits straightforward referencing and analysis over temporal intervals. Furthermore, the dataset is compatible with machine learning methodologies, particularly in the context of training object detection and segmentation algorithms to evaluate environmental transformations.

### 4.2 Image enhancement and annotation

The annotation process for this dataset was carried out using the AI tool Make Sense, which allowed for precise labeling of satellite images. In this process, trees and deforested areas were manually marked by drawing bounding boxes around each area of interest. These bounding boxes were carefully created to capture the specific regions containing tree cover and areas that had been cleared, ensuring that each labeled area was accurately represented.

**Fig. 5:**
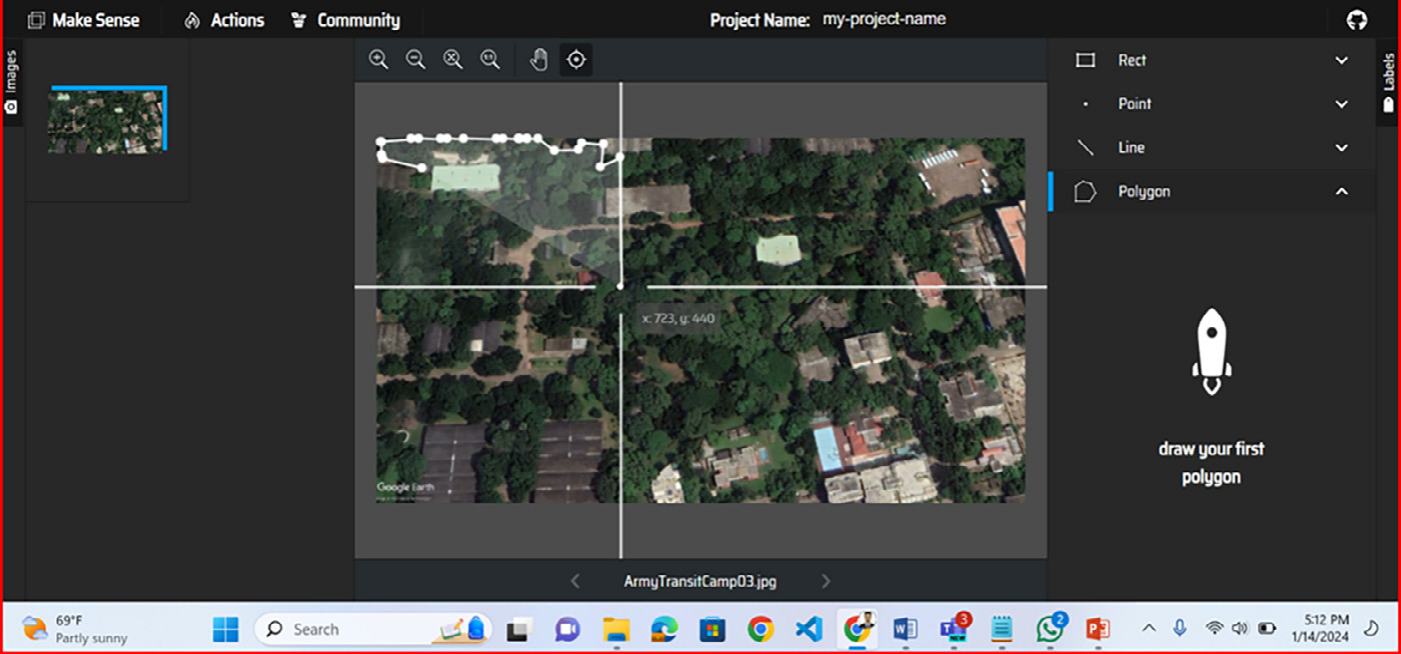
Sample of image annotating with make sense AI picture.

**Fig. 6:**
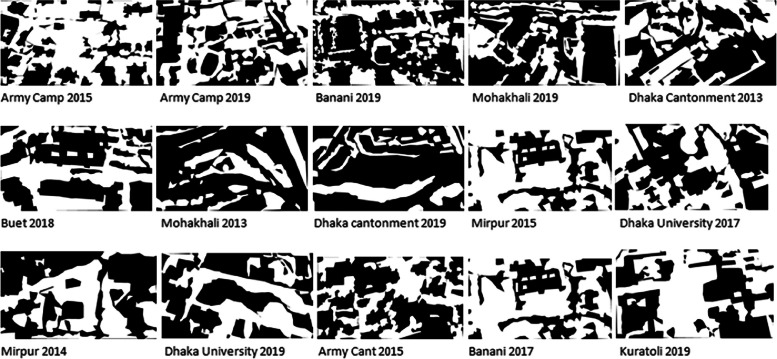
Sample of mask picture.

This manual approach enabled high-resolution, pixel-level annotations, which are crucial for training machine learning models effectively. Once the bounding boxes were drawn and labeled, they were converted into segmentation masks. These masks provide an outline of the specific regions where trees and deforested areas are located, translating the bounding box annotations into a format suitable for machine learning tasks like semantic segmentation, object detection, and change detection. The masks highlight individual pixels within the images that correspond to tree cover and deforested regions, allowing machine learning models to learn the spatial characteristics of each category. To ensure compatibility and easy integration with various machine learning frameworks, these masks were saved in JSON format. The JSON files contain detailed information about the coordinates and dimensions of each bounding box and the mask’s pixel locations. This structured format allows for seamless processing within machine learning pipelines, enabling researchers to feed the data directly into different models for training and analysis. The JSON annotations can also be easily manipulated or expanded upon for additional tasks, providing a flexible data structure that supports a wide range of machine learning applications in environmental monitoring and deforestation analysis. This careful and structured annotation process enhances the accuracy of models trained on this dataset, contributing to robust deforestation analysis and conservation efforts. Fig. 5 illustrates a sample of image annotation using the Make Sense AI tool, showcasing the bounding boxes drawn around areas of interest. Fig. 6 provides a sample of the resulting mask picture, highlighting the pixel-level segmentation of tree cover and deforested areas.

### 4.3 Data pre-processing

Various image enhancement techniques such as random cropping, image rotation and contrast adjustment were applied to improve the dataset. The enhanced images were then divided into training, test and validation sets to create more reliable models for machine learning.

### 4.4 Image segmentation

The masked images were used for image segmentation tasks to identify deforested areas to support the training of machine learning models that can accurately track environmental changes.

### 4.5 Dataset evaluation

**Fig. 7:**
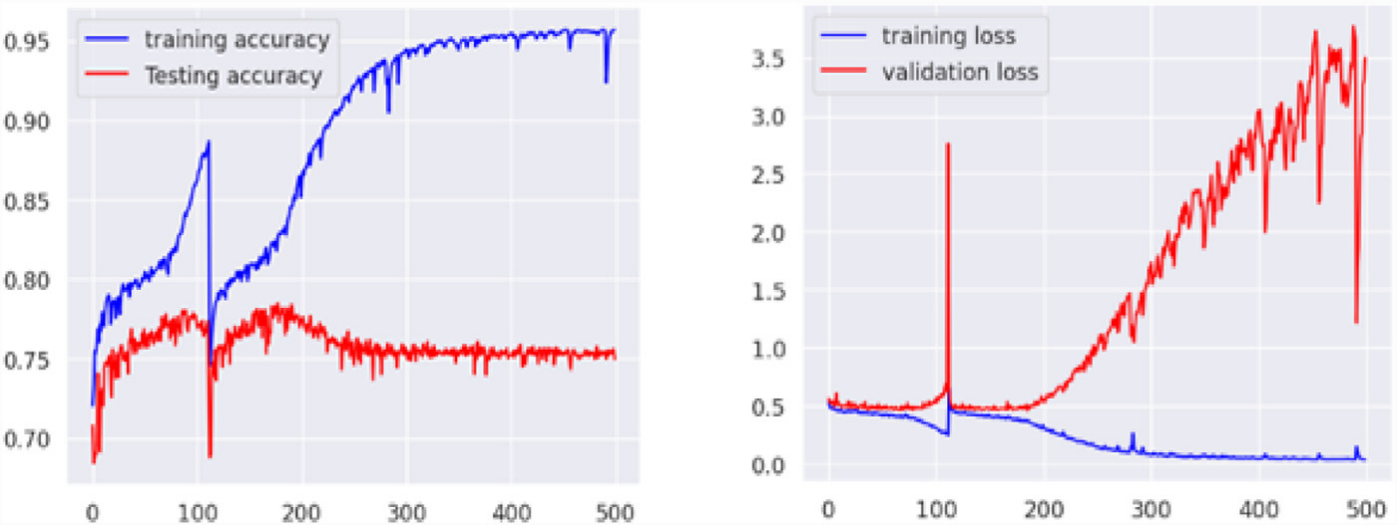
Accuracy and loss of U-Net model.

**Fig. 8:**
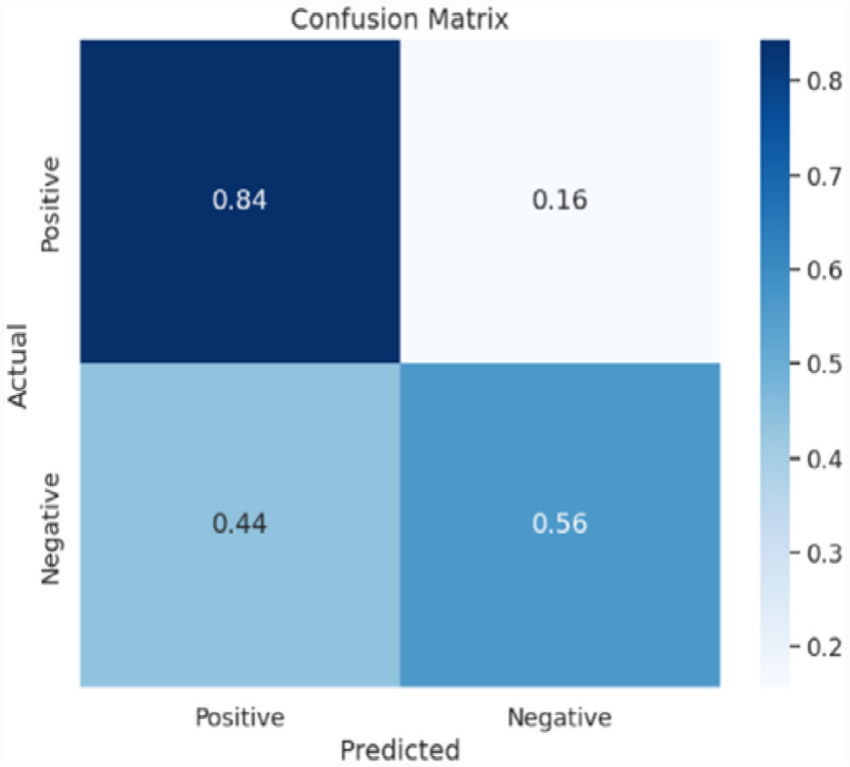
Confusion matrix of U-Net model.

**Fig. 9:**
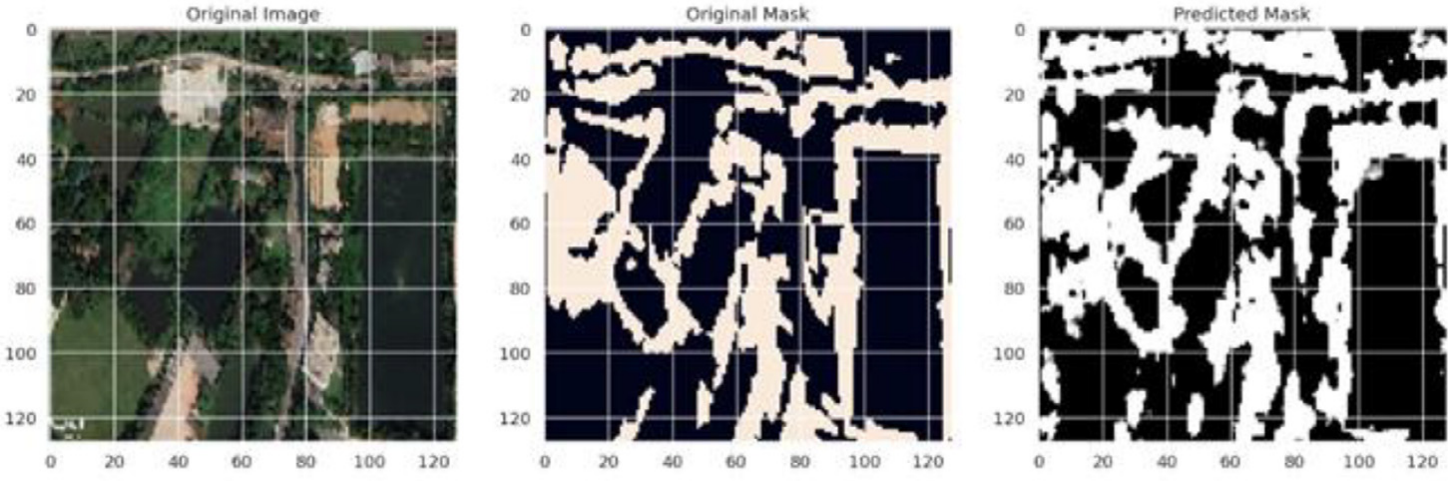
Predicted mask of U-Net.

The dataset was analysed using machine learning algorithms, including object detection models for tree cover analysis and deforestation tracking. Pre-built models were tested to ensure that the data supported accurate predictions and reliable segmentation. Evaluation metrics, such as mean average precision (mAP), highlighted the effectiveness of the dataset in capturing deforestation trend. (Fig. 7, Fig. 8, Fig. 9).

The U-Net model underwent a training process consisting of 500 epochs and demonstrated good performance with the dataset. Throughout the validation, the model achieved a high loss, indicate that the model is not performing well on the given task. After completing the 500 epochs, the model's validation accuracy reached 76%, while the training accuracy stood at 95.66%. These high accuracy and loss rates indicate that the model was not able to accurately classify the data.

To further evaluate the model's performance, a confusion matrix was generated. The confusion matrix provides insights into the model's predictive abilities by presenting the distribution of correct and incorrect predictions across different year. The results of the confusion matrix for U-Net showed a recall and F1 score of 76% each, which means that the model was able to correctly identify the positive and negative instances with high accuracy. Additionally, the overall accuracy of the model, as reflected in the confusion matrix, was 77% (Table 1).

**Table 1:** Dataset evaluation on U-Net model.

F1 Score	Precision	Recall	Accuracy
76%	77%	76%	77%

### 4.6 Performance analysis

In this study, the segmentation results are visually depicted through a sequence of images, encapsulating the essence of the model's performance. The original image, serving as the input to the segmentation model, is showcased in original image, providing a contextual understanding of the visual content under examination. Subsequently, original mask displays the mask image, representing the manually annotated segmentation, serving as the reference for evaluating the model's accuracy. The predicted mask, generated by the trained segmentation model. A visual comparison between the mask image and the model's prediction is discussed, revealing insights into the model's proficiency in delineating object boundaries. This evaluation is complemented by quantitative metrics, with the model achieving a Dice coefficient of Y%, providing a numerical foundation for assessing the segmentation accuracy.

## Limitations

These limitations provide important context for understanding the constraints of the dataset and potential impacts on model performance and generalizability:

• The dataset is restricted to Dhaka, Bangladesh, focusing on an urban environment rather than rural or forested regions. This narrow geographic scope may limit its ability to generalize deforestation trends across different landscapes, ecosystems, or other regions of Bangladesh.
• While the dataset includes high-resolution images, variability in image quality due to lighting, cloud cover, and atmospheric conditions across different years introduces potential inconsistencies. These variations can reduce the accuracy and reliability of models trained on this data.
• The dataset is based on annual satellite imagery, which may not capture short-term or seasonal changes in deforestation. As a result, rapid tree losses or recoveries within a year may not be fully captured, which may limit the temporal resolution of the analysis.
• Since the labelling of tree cover and deforested areas was done manually, human error or subjective interpretation may lead to inconsistencies in the labelled data. This could impact the performance of machine learning models trained on the dataset.
• While some field validation was conducted, it may not have been possible to physically verify all locations. This could lead to discrepancies between the satellite image interpretation and the actual conditions on the ground, especially in dense urban areas where it can be more difficult to accurately assess tree cover.
• The number of images (5-35 per site per year) may not be sufficient to capture all variations in deforestation patterns, especially for deep learning models that require large datasets for optimal performance. Increasing the amount of data is helpful but cannot fully compensate for the limited size of the dataset.
• As Dhaka is a densely populated urban area, factors such as construction of buildings, expansion of roads and other urban activities could be mistaken for deforestation, which could lead to contamination of the dataset. Distinguishing between natural tree loss and urban development can be a challenge.

## Ethics Statement

The authors declare that the hereby presented data and data article fully comply with the Journal’s policy in terms of authors’ duties, data integrity, and experimental requirements.

## CRediT Author Statement

**Md Fahad Khan**: Formal analysis, Validation, Methodology, Writing – original draft; **Md Rakibul Islam**: Data curation, Conceptualization, Resources, Writing – original draft; **Shanto Kumar Basak:** Writing - Original draft; **Ahmed Imtiaz:** Writing - Original draft, Background & Editing; **Abhijit Bhowmik**: Writing - Original draft; **Dip Nandi**: Writing – review & editing; **Mashiour Rahman**: Validation, Writing – review & editing; **Debajyoti Karmaker**: Supervision, Writing – review & editing.

## Data Availability

Mendeley DataGeospatial Dataset on Deforestation and Urban Sprawl in Dhaka, Bangladesh: A Resource for Environmental Analysis (Original data)
